# Differential Effects of Rotatable Non-Host Crop Cultivation Duration on Physicochemical and Microbial Properties of Monocropping Soil

**DOI:** 10.3390/plants15192983

**Published:** 2026-09-30

**Authors:** Xianfu Yuan, Dan Wang, Xueli Zhang, Jianfei Wang

**Affiliations:** 1 College of Resources and Environment, Anhui Science and Technology University, Chuzhou 233100, China; yuanxf@ahstu.edu.cn (X.Y.); 18365326365@163.com (D.W.); 15556383285@163.com (X.Z.); 2 Anhui Provincial Engineering Research Center for Fertilizer Utilization of Agricultural Wastes and Cultivated Land Quality Improvement, Bengbu 233400, China

**Keywords:** banana continuous cropping, single-season rotation, long-term monocropping of rotatable non-host crop, soil-borne pathogens, soil microorganisms, soil physical and chemical properties, soil enzyme activity

## Abstract

Crop rotation effectively mitigates soil continuous cropping obstacles. Exploring the impacts of different cropping systems (rotational cultivation vs. long-term monocropping of rotatable non-host crop) on the physicochemical (including pH, electrical conductivity, soil organic matter, available phosphorus, available potassium, ammonium nitrogen, and nitrate nitrogen) and microbial properties (including the abundances of *Fusarium oxysporum* f. sp. *cubense* (FOC) total bacteria, fungi, and actinomycetes, as well as catalase, urease, and chitinase activities) of monocropped soil is essential for targeted obstacle management. In a long-term fixed-site experiment, three treatments were established: continuous banana monocropping (BB), single-season pineapple rotation (BP), and long-term pineapple monocropping after continuous banana monocropping (PP). Soil physicochemical and microbial properties were determined, and their interrelationships were analyzed. Compared with BB, both BP and PP significantly reduced the target gene copies of FOC with the FOC target gene copies in BP being significantly (*p* < 0.05) higher than in PP. BP significantly (*p* < 0.05) increased the target gene copies of fungi (F), bacteria (B) and actinomycetes (A) as well as the activities of catalase (Cat), urease (Ure) and chitinase (Chi). In contrast, PP caused no significant changes in actinomycetes’ target gene copies or any tested enzyme activity. All of the microbial target gene copies and enzyme activities in BP were significantly (*p* < 0.05) higher than those in PP. Relative to BB, BP significantly (*p* < 0.05) decreased the soil available potassium (SK), available phosphorus (SP) and soil organic matter (SOM). Soil pH and SOM were significantly (*p* < 0.05) higher in BP than in PP, while electrical conductivity (EC) and nitrate nitrogen (NO_3_^−^) were significantly (*p* < 0.05) higher in PP than in BB. Model analysis indicated that SOM, SK, SP and NH_4_^+^-N were the core physicochemical factors explaining the variation in FOC target gene copies, and the target gene copies of actinomycetes and fungi, together with catalase, urease and chitinase activities, were the key biological drivers. Verification tests showed that the proportions of pathogen-antagonistic fungi and actinomycetes in BP reached 23.6% and 10%, respectively, which were significantly (*p* < 0.05) higher than those in PP and BB. Banana seedlings grown in BP soil had significantly (*p* < 0.05) greater plant height, stem diameter and leaf area than those in BB. PP showed a mild upward trend in growth indicators, but the difference from BB was not significant. In conclusion, single-season pineapple rotation and the long-term monocropping of rotatable non-host crop both reduce FOC target gene copies in continuously cropped soil, yet the two patterns operate through distinct suppression mechanisms for continuous cropping obstacles. Single-season rotation achieves better improvement in soil physicochemical and microbial properties, while the long-term monocropping of a rotatable non-host crop showed lower soil FOC target gene copies.

## 1. Introduction

Cropping obstacles are the problems that arise when the same crop is grown repeatedly in the same field for many years. This causes the deterioration of physical and chemical conditions in the soil, an increased accumulation of harmful disease-causing microbes, and a much higher occurrence of crop diseases [1], among which Fusarium spp. are widely recognized as the main cause of such soil-borne crop diseases [1]. Banana Fusarium wilt is a soil-borne disease caused by *Fusarium oxysporum* f. sp. *cubense* (FOC). Once infecting plant roots, the pathogen induces vascular-bundle blockage and can even kill the entire plant [2], posing grave threats to the global banana industry [2]. When bananas are planted continuously in the same soil for many years, FOC gradually accumulates in the soil over time, and control becomes increasingly difficult because this pathogen spreads in many different ways and possesses strong pathogenicity [3]. Currently, regulating soil microbial communities is considered an effective approach to managing Fusarium wilt disease [4].

At present, Fusarium wilt is managed mainly through the application of organic fertilizers [5], soil fumigation [6], the breeding of disease-resistant cultivars [7], and crop rotation [8]. Among these, organic fertilizers can improve soil microbial communities and enzyme activity, yet their application may also introduce harmful pathogenic microbes and heavy metals into the soil environment [9]. Soil fumigation shows rapid disease control, but it also causes severe damage to the ecological health of the soil and leaves large amounts of chemical residues, which does not align with the goals of developing green and sustainable agriculture [10]. Breeding disease-resistant crop varieties can reduce disease incidence from the source, but the whole process of breeding high-quality resistant varieties is very time consuming [3]. Some of these new resistant varieties may also carry undesirable agricultural traits or produce fruit of lower quality [11]. Crop rotation, by comparison, is more environmentally friendly: pathogen populations decline, beneficial microbes become more abundant, and autotoxic substances already present in the soil are degraded or transformed [8], so that the constraints associated with continuous cropping can be alleviated [12]. Two mechanisms are generally considered to underlie this suppression of soil-borne diseases. The first is straightforward: once a non-host crop enters the rotation, the pathogen loses access to the host it needs for survival, and its population becomes difficult to maintain [13]. The second relates to the soil microbial community. Rotation improves the community structure in continuously cropped soil and raises microbial diversity [13], and the distinct root systems of successive crops can further inhibit continuous pathogen proliferation, slow the rate of disease spread, and in some cases even improve soil physical structure [13]. It should be noted, however, that the disease-suppressive performance varies considerably with rotation duration and with the crop species chosen [14]. Previous studies have found that rotating with cassava for one to three years provided significant control of banana Fusarium wilt [15]; rotating with non-cruciferous crops for more than five years reduced soil pathogen abundance [16], and rotating for more than eight years before planting Coptis chinensis proved highly effective against root rot disease [17]. Our team has established a new rotation system for pineapple and banana, and a single-season application of this banana–pineapple rotation system shows significant effects on controlling banana *Fusarium* wilt [18]. A mechanistic analysis reveals that pineapple rotation reshapes soil microbial community composition. It especially helps enrich antagonistic fungal taxa that fight against the pathogen, which then stop the pathogen from growing and reduce disease incidence. At present, how the cropping duration of rotation-suitable non-host crops differentially influences the mitigation of continuous cropping obstacles and the shifts in soil pathogen populations remains poorly understood. In particular, the distinct regulatory mechanisms underlying pathogen suppression between single-season rotation and the long-term monocropping of rotatable non-host crops also remain unclear.

This paper takes banana continuous cropping *Fusarium* wilt as the research object and adopts a banana–pineapple rotation system. The selection of pineapple as the rotation crop is grounded in host specificity: *Fusarium oxysporum* f. sp. *cubense* (FOC) is specific to *Musa* spp., whereas pineapple is affected by taxonomically distinct *Fusarium* species (*F. guttiforme*/*F. ananatum*), thereby breaking the banana-specific pathogen cycle—a key mechanistic premise of this rotation strategy. We analyzed the effects of single-season banana–pineapple rotation (13 months) and long-term pineapple monocropping (more than 5 years) on the pathogen abundance of banana monocropping soil, microbial population size, enzyme activities, soil physicochemical properties, and the proportion of antagonistic microorganisms against pathogenic microbes. This paper further explores the differential control efficacies of banana–pineapple rotation and long-term pineapple monocropping on continuous cropping obstacles with the aim of providing a theoretical basis for the rational arrangement of consecutive cropping seasons of rotation-suitable non-host crops within banana planting systems.

## 2. Materials and Methods

### 2.1. Experimental Site Description

The field experiment was conducted in Jianfeng Town, Ledong Li Autonomous County, Hainan Province, China (geographical coordinates: 18°42′ N, 108°49′ E), where a long-term banana–pineapple rotation experiment has been maintained since 2014. The region has a typical tropical monsoon climate, with an annual average temperature of 23–25 °C and annual average precipitation of 1600–1800 mm, which is characterized by concurrent rainfall and high temperature. The soil type of the experimental field is tropical latosol with initial basic physicochemical properties as follows: soil organic matter content 12.72 g/kg, total nitrogen content 0.61 g/kg, total phosphorus content 0.48 g/kg, and total potassium content 6.71 g/kg. All determinations were performed according to the standard methods described in Soil and Agricultural Chemistry Analysis (Bao, 2000) [19].

### 2.2. Experimental Design

The experiment was carried out from August 2024 to November 2025 with three cropping regime treatments established: BB (Continuous banana monocropping): plots under continuous banana cultivation for more than 5 years; BP (Single-season pineapple rotation): plots previously under continuous banana monocropping for >5 years followed by one 13-month season of pineapple rotation; PP (long-term pineapple monocropping after continuous banana monocropping): plots previously under continuous banana monocropping for >5 years, followed by five consecutive 13-month pineapple monocropping systems, with a total cultivation duration exceeding 5 years. The experiment was designed around the banana–pineapple rotation practices that dominate in Hainan, China. Pineapple there has a growth cycle of about 13 months, and after conversion from diseased banana fields, farmers usually keep pineapple for multiple seasons. Our previous study showed that single-season pineapple rotation can suppress banana Fusarium wilt [18]; accordingly, the BB, BP, and PP treatments were set up to represent the most common local rotation regimes, which allowed short-term and long-term rotation effects to be compared. Three randomly arranged plots, each covering 60 m^2^ (3 m in width and 20 m in length), were used as replicates for every treatment. Fertilization and irrigation were identical for all treatments: over the entire banana growth period, total nitrogen (N), phosphorus (P), and potassium (K) were applied at 160 kg N·hm^−2^, 30 kg P_2_O_5_·hm^−2^, and 225 kg K_2_O·hm^−2^, respectively. The remaining agronomic practices were likewise kept the same among the three treatments—drip irrigation, monthly manual weeding without herbicides, and pest and disease control following the same fungicide and insecticide schedule and dosage. Banana plots all received standard management, namely sucker removal, bamboo propping, and fruit bagging, while pineapple plots were managed uniformly with respect to planting density, flower forcing, and harvesting.

### 2.3. Soil Sample Collection and Processing

Soil sampling was conducted in November 2025 across all treatment plots. Soil physicochemical and microbial properties are known to be spatially heterogeneous in field experiments [20]. To account for such within-plot micro-environmental variation, three spatially independent composite samples were taken from distinct micro-sites along an S-shaped transect in each plot with neighboring sampling sites at least 5 m apart. Each composite sample consisted of five randomly collected soil cores from the 0–20 cm plow layer, which were thoroughly homogenized before analysis. This yielded a total of 9 independent composite samples (3 treatments × 3 replicate plots), which served as the unified material basis for all subsequent soil physicochemical, enzymatic, and molecular (qPCR) determinations. Immediately after collection, samples were transported to the laboratory in insulated coolers maintained at 4 °C. Each composite sample was further divided into three subsamples under sterile conditions: one subsample was stored at 4 °C for culturable microbial isolation, screening, and soil enzyme activity assays; a second subsample was stored at −80 °C for a quantitative polymerase chain reaction (qPCR) analysis of soil microbial populations; the third subsample was air-dried in a cool, well-ventilated area, ground, and passed through a sieve for the subsequent determination of soil physicochemical properties.

### 2.4. Determination of Soil Samples

#### 2.4.1. Extraction of Total Microbial DNA and Quantitative PCR

Total genomic DNA was isolated from soil samples of each treatment using the PowerSoil DNA Isolation Kit (Qiagen, Hilversum, The Netherlands). The target gene copies of total bacteria, fungi, and actinomycetes in soil were quantified by real-time quantitative PCR on an ABI 7500 system with SYBR Green qPCR Master Mix (SparkJade Biotechnology Co., Ltd., Qingdao, China) employed for fluorescence detection [21]. The quantitative real-time PCR (qPCR) was run on an ABI 7500 instrument (Applied Biosystems, Foster City, CA, USA) to quantify the gene copy numbers of total fungi, bacteria and actinomycetes. Three primer pairs (synthesized by Tsingke Biotechnology Co., Ltd., Beijing, China) were used for targeted amplification: the ITS1/5.8S pair (forward: 5′-TCCGTAGGTGAACCTGCGG-3′; reverse: 5′-CGCTGCGTTCTTCATCG-3′) for fungal communities, the 357/518 pair (forward: 5′-TACGGGAGGCAGCAG-3′; reverse: 5′-ATTACCGCGGCTGCTGG-3′) for bacterial communities, and the 243F/1387R pair (forward: 5′-GGATGAGCCCGCGGCCTATCAG-3′; reverse: 5′-GGGCGGWGTGTACAAGGC-3′) for actinomycetes. FOC (*Fusarium oxysporum* f. sp. *cubense*) quantification was carried out following the method established by Huang et al. [22]. The thermal cycling procedure included an initial denaturation at 95 °C for 30 s, 30 cycles of denaturation at 95 °C for 5 s and annealing/extension at 60 °C for 34 s, which was followed by a melting curve stage with incubation at 95 °C for 15 s, 60 °C for 1 min, and 95 °C for 15 s. Each 20 µL reaction system contained 10 µL of 2× SYBR Premix, 0.4 µL of each forward and reverse primer, 0.4 µL of 50× ROX II reference dye, 2 µL of template DNA, and 6.8 µL of double-distilled water.

#### 2.4.2. Determination of Soil Physicochemical Properties

Soil pH was measured using the potentiometric method with a pH electrode (PHS-3C, Shanghai INESA Scientific Instrument Co., Ltd., Shanghai, China), and soil pH was measured with a soil–water ratio of 1:2.5 (*w*/*v*). Soil electrical conductivity (EC) was determined using the electrode method with a conductivity meter (DDS-307, Shanghai INESA Scientific Instrument Co., Ltd., Shanghai, China), and electrical conductivity (EC) was determined at a soil–water ratio of 1:5 (*w*/*v*). The soil organic matter (SOM) content was quantified via the potassium dichromate external heating oxidation–titration method. Available potassium (AK) was extracted with ammonium acetate and measured by flame photometry (FP6410, Shanghai INESA Analytical Instrument Co., Ltd., Shanghai, China). The available phosphorus (AP) was determined using the 0.5 mol·L^−1^ NaHCO_3_ extraction method. Nitrate nitrogen (NO_3_^−^) and ammonium nitrogen (NH_4_^+^) were measured via ultraviolet spectrophotometry(UV-5100, Shanghai Metash Instruments Co., Ltd., Shanghai, China) and the potassium chloride extraction-indophenol blue colorimetric method, respectively. All analytical procedures followed the standard methods described in Soil Agrochemical Analysis [19]. All chemical reagents used in the above determinations were of analytical grade (Sinopharm Chemical Reagent Co., Ltd., Shanghai, China).

#### 2.4.3. Determination of Soil Enzyme Activities

All soil enzyme activities were determined using commercially available enzyme activity assay kits (Boxbio, Beijing, China), following the manufacturer’s instructions. Enzyme activity was expressed as the amount of substrate converted per unit mass of soil per unit time with the unit of U·g^−1^·d^−1^.

### 2.5. Determination of the Proportion of Pathogen-Antagonistic Fungi and Actinomycetes

For each treatment, isolates from the nine corresponding composite samples were pooled to obtain a total of 100 culturable fungal isolates and 100 culturable actinomycetal isolates per treatment, ensuring that the screened isolates represented the full spatial heterogeneity of each treatment rather than a single sampling point. Fungi were isolated on potato dextrose agar (PDA) medium (Qingdao Hope Bio-Technology Co., Ltd., Qingdao, China), and actinomycetes were isolated on Gause’s No. 1 synthetic medium (Qingdao Hope Bio-Technology Co., Ltd., Qingdao, China) [22]. After three successive rounds of purification, the antagonistic activity of each isolate against *Fusarium oxysporum* f. sp. *cubense* (FOC), the causal agent of banana *Fusarium* wilt, was determined via the dual-culture plate assay [23]. The number of isolates with significant antagonistic effects was counted, and the proportion of antagonistic isolates relative to the total number of tested isolates was calculated.

### 2.6. Pot Experiment and Determination of Banana Growth Indicators

A pot validation experiment was conducted in the greenhouse of Anhui Science and Technology University in April 2026. Bulk soil from the 0–20 cm plow layer was collected separately from the three field experimental treatments: multi-season pineapple rotation (PP), single-season pineapple rotation (BP), and continuous banana monocropping (BB). Soil from each treatment was thoroughly homogenized, and 1 kg of the homogenized soil was placed into each plastic pot with nutrient levels adjusted to a uniform baseline across all treatments to eliminate nutrient variation as a confounding factor. The banana cultivar used in the experiment was *Musa acuminata* AAA *Cavendish* cv. Brazilian. Banana seedlings with uniform growth vigor and no visible symptoms of disease or pest damage were selected and transplanted into the pots with one seedling per pot. All treatments received identical water and fertilizer management throughout the experiment. Two months after transplanting, when distinct phenotypic differences in plant growth (e.g., plant height, stem diameter, and leaf area) between treatments were observed, three core growth indices were measured: plant height, stem diameter, and leaf area. Plant height was measured using a measuring tape as the vertical distance from the plant base to the bifurcation of the two newest leaf petioles. Stem diameter was measured using a vernier caliper (Shanghai Measuring and Cutting Tool Works Co., Ltd., Shanghai, China) at the position of 2 cm above the soil surface on the pseudostem. For leaf area determination, three fully expanded leaves from the upper, middle, and lower positions of each plant were selected, and the leaf length (L) and maximum leaf width (W) were measured [24].

### 2.7. Data Processing

Raw data were initially organized using Microsoft Excel 2023 (Microsoft Corporation, Redmond, WA, USA). Graph plotting and statistical analysis were performed using Origin 2021 (OriginLab Corporation, Northampton, MA, USA) and IBM SPSS Statistics 26 (IBM Corporation, Armonk, NY, USA), respectively. A one-way analysis of variance (ANOVA) was used to assess differences among group means, and all pairwise comparisons were subsequently conducted using Tukey’s Honestly Significant Difference (HSD) test at a significance level of *p* < 0.05. For correlation analyses, Spearman’s rank correlation coefficient was used to assess the relationships between variables, while the Benjamini–Hochberg false discovery rate (FDR) correction was applied to adjust *p*-values and control for multiple comparisons. Multiple linear regression was then used to model FOC target gene copies with the seven soil physicochemical factors entered simultaneously in one model and the six microbial and enzyme factors entered simultaneously in a second model. The relative importance of each predictor was quantified using the LMG metric, which averages the incremental R^2^ contribution of each variable over all possible predictor orderings. For both models, residual normality was examined using the Shapiro–Wilk test, and effects were considered significant at *p* < 0.05.

## 3. Results

### 3.1. Effects of Different Pineapple Cropping Regimes on the Abundances of Pathogens, Fungi, Bacteria, and Actinomycetes

**Figure 1:**
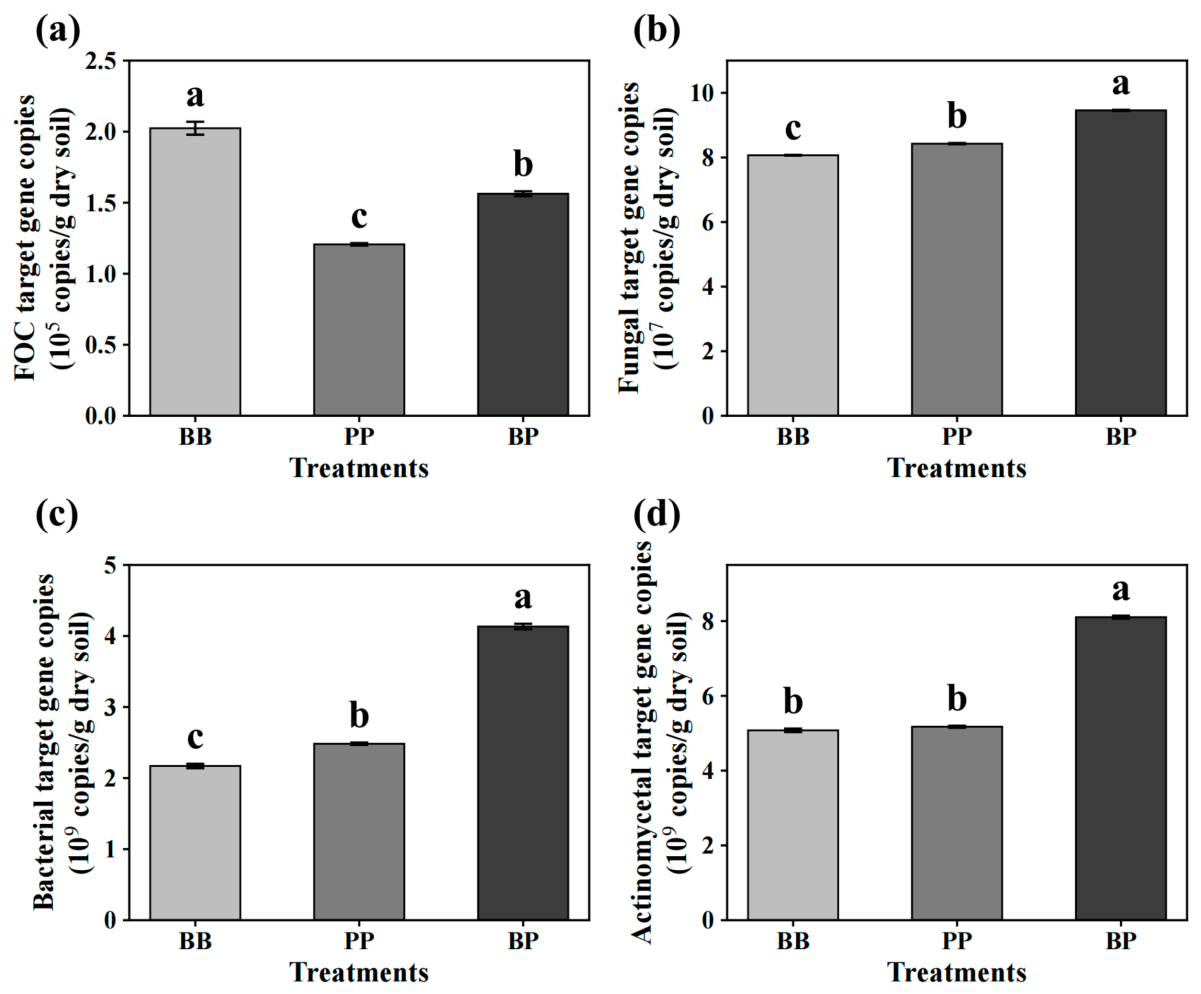
Effects of different cropping patterns on soil microbial gene copies ((**a**) FOC, (**b**) Bacteria, (**c**) Fungi, (**d**) Actinomycetes). Note: Error bars represent standard errors (SEs). The lowercase letters marked in the figure indicate significant differences among different treatments at the *p* < 0.05 level based on Tukey’s HSD test; the same applies below. BB: Continuous banana monocropping; BP: Single-season pineapple rotation; PP: Long-term pineapple monocropping after continuous banana monocropping.

As shown in Figure 1a, compared with the continuous banana monocropping (BB) treatment, both the long-term pineapple monocropping (PP) and single-season pineapple rotation (BP) treatments significantly (*p* < 0.05) reduced the target gene copies of *Fusarium oxysporum* f. sp. *cubense* (FOC) with the target gene copies of FOC in BP being significantly (*p* < 0.05) higher than that in PP. As illustrated in Figure 1b,c, the fungal and bacterial target gene copy numbers in both the BP and PP treatments were significantly (*p* < 0.05) higher than those in the BB treatment; moreover, the fungal and bacterial gene copy numbers in the BP treatment were significantly (*p* < 0.05) higher than those in the PP treatment. As presented in Figure 1d, compared with BB, the BP treatment significantly (*p* < 0.05) increased the target gene copies of actinomycetes, while no significant difference in the target gene copies of actinomycetes was observed between PP and BB. Additionally, the target gene copies of actinomycetes in BP was significantly (*p* < 0.05) higher than that in PP.

### 3.2. Effects of Different Pineapple Cropping Regimes on Soil Physicochemical Properties

**Figure 2:**
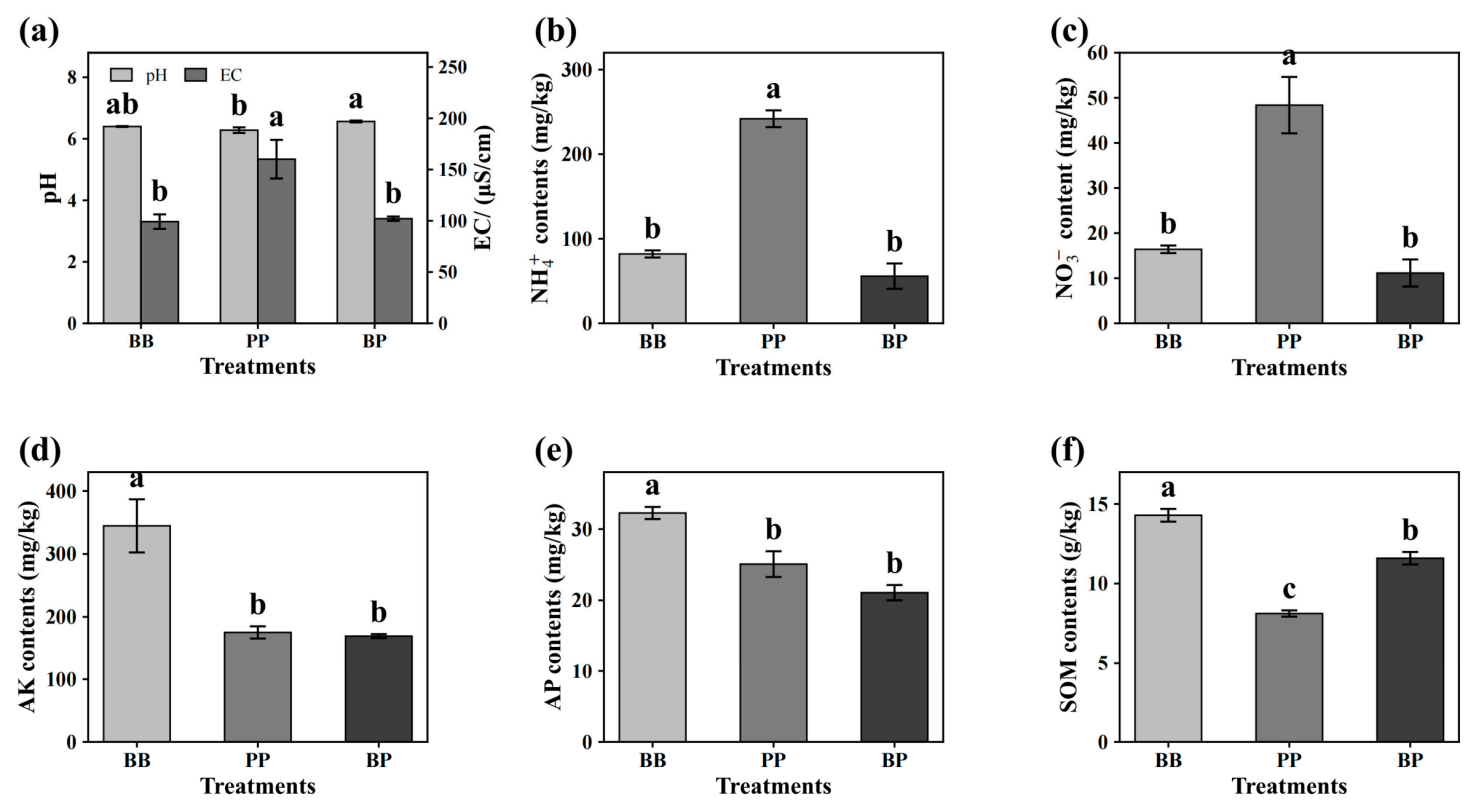
Effects of different cropping systems on soil physicochemical properties: (**a**) soil pH and electrical conductivity (EC); (**b**) ammonium nitrogen (NH_4_^+^); (**c**) nitrate nitrogen (NO_3_^−^); (**d**) available potassium (AK); (**e**) available phosphorus (AP); and (**f**) soil organic matter (SOM). Note: Error bars represent standard errors (SEs). The lowercase letters marked in the figure indicate significant differences among different treatments at the *p* < 0.05 level based on Tukey’s HSD test; the same applies below. BB: Continuous banana monocropping; BP: Single-season pineapple rotation; PP: Long-term pineapple monocropping after continuous banana monocropping.

As shown in Figure 2a, compared with the BB treatment, soil pH showed no significant (*p* < 0.05) change in either BP or PP treatments, while the pH in PP was significantly (*p* < 0.05) higher than that in BP. Meanwhile, soil electrical conductivity (EC) remained unchanged in BP relative to BB but was significantly (*p* < 0.05) increased in the PP treatment. In addition, compared with BB, the PP treatment showed significantly (*p* < 0.05) higher ammonium nitrogen (NH_4_^+^-N) content (Figure 2b), while available potassium (AK, Figure 2d), available phosphorus (AP, Figure 2e), and soil organic matter (SOM, Figure 2f) contents were significantly (*p* < 0.05) reduced. For the PP treatment, both nitrate nitrogen (NO_3_^−^-N, Figure 2c) and NH_4_^+^-N (Figure 2b) contents were significantly (*p* < 0.05) higher than those in BB, whereas AP, AK, and SOM contents were significantly (*p* < 0.05) lower. Further comparisons between the two rotation treatments revealed that the NO_3_^−^ and NH_4_^+^ contents in BP were significantly (*p* < 0.05) lower than those in PP, while the SOM content was significantly (*p* < 0.05) higher in BP than in PP. No significant differences in AP and AK contents were observed between BP and PP treatments.

### 3.3. Effects of Different Pineapple Cropping Regimes on Soil Enzyme Activities

**Figure 3:**
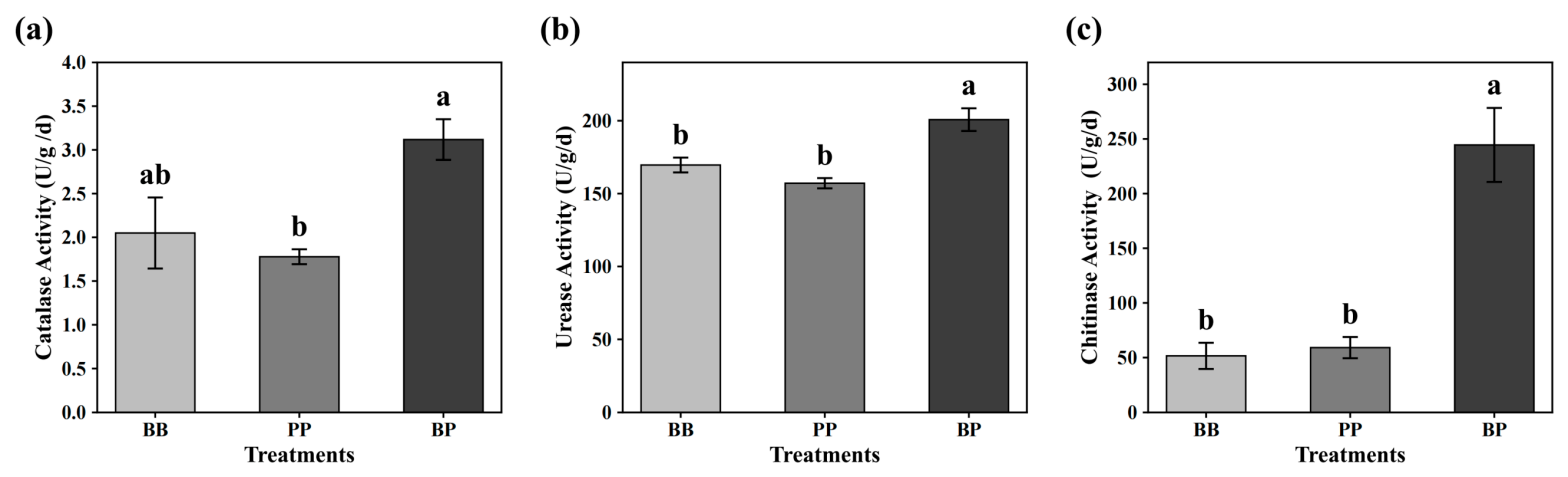
Effects of different cropping patterns on soil enzyme activities ((**a**) Catalase, (**b**) Urease, (**c**) Chitinase). Note: Error bars represent standard errors (SEs). The lowercase letters marked in the figure indicate significant differences among different treatments at the *p* < 0.05 level based on Tukey’s HSD test, and the same applies below. BB: Continuous banana monocropping; BP: Single-season pineapple rotation; PP: Long-term pineapple monocropping after continuous banana monocropping.

As illustrated in Figure 3a, compared with the BB treatment, catalase (CAT) activity was significantly (*p* < 0.05) increased in the BP treatment but significantly (*p* < 0.05) decreased in the PP treatment. The catalase activity in BP was significantly (*p* < 0.05) higher than that in PP. As shown in Figure 3b, urease (URE) activity was significantly (*p* < 0.05) higher in the BP treatment than in BB, while no significant difference in urease activity was observed between PP and BB. The urease activity in BP was also significantly (*p* < 0.05) higher than that in PP. As presented in Figure 3c, chitinase (CHI) activity was significantly (*p* < 0.05) increased in the BP treatment relative to BB but significantly (*p* < 0.05) decreased in the PP treatment. Chitinase (CHI) activity in BP was significantly (*p* < 0.05) higher than that in PP.

### 3.4. Correlation Analysis Between Soil Pathogen Abundance and Soil Physicochemical Properties, Enzyme Activities, and Microbial Abundances

**Figure 4:**
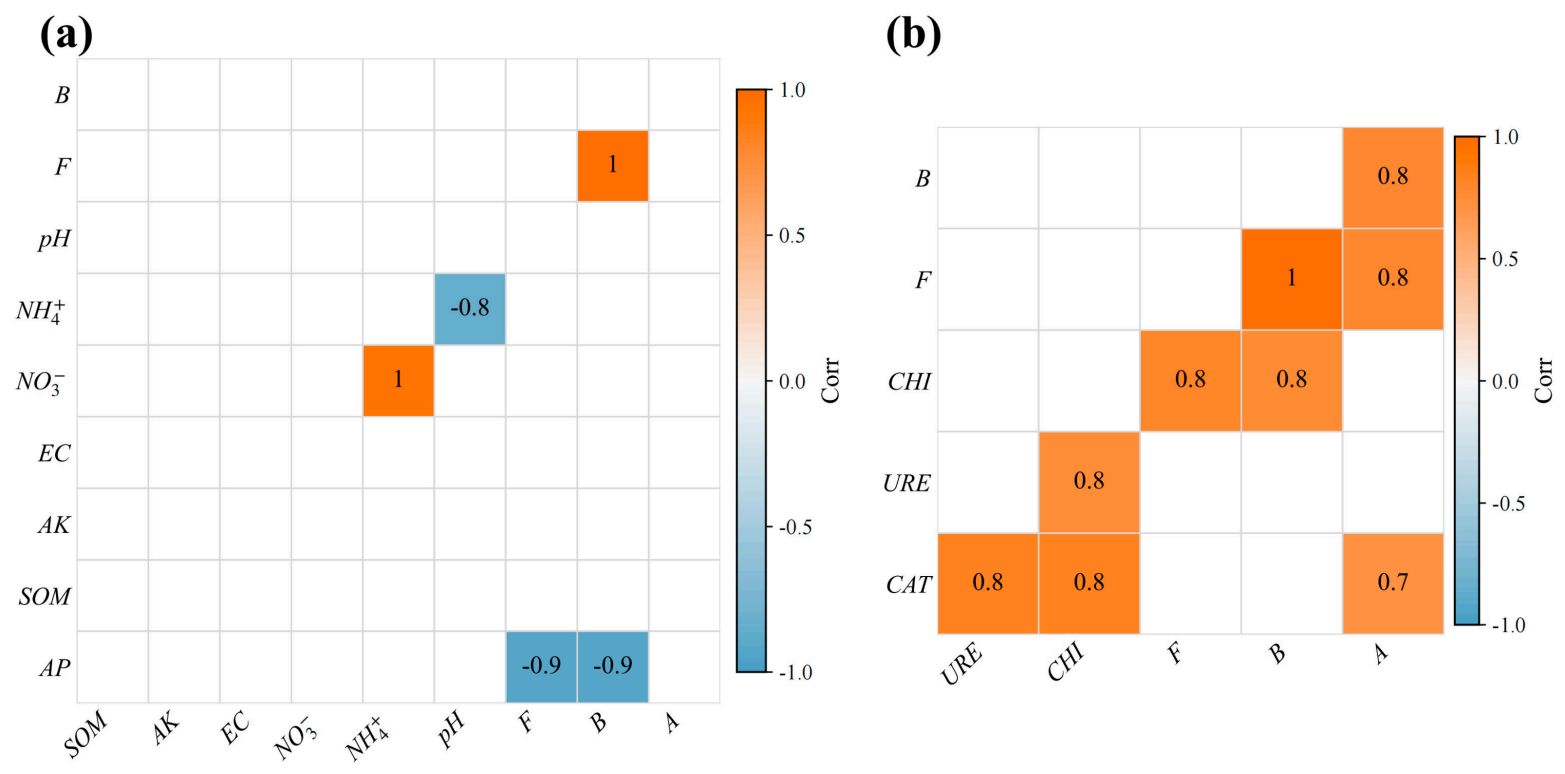
Correlation analysis of soil physicochemical properties, enzyme activities, and microbial abundance. (**a**) Correlation between soil physicochemical properties and microbial abundance; (**b**) Correlation between soil enzyme activities and microbial abundance. Notes: The colored squares marked in the figure indicate significant correlation among different parameters at the *p* < 0.05 level. The *p* values were adjusted by the Benjamini–Hochberg method to control the false discovery rate (FDR).

As shown in Figure 4a, soil fungal target gene copies were significantly (*p* < 0.05) and negatively correlated with soil available phosphorus (AP) with a correlation coefficient of r = −0.9, whereas their negative association with available potassium (AK) was not significant. Bacterial target gene copies also showed a significant (*p* < 0.05) negative correlation with AP (r = −0.9); the negative trends of actinomycete target gene copies with AP and AK did not reach significance. In contrast, the three microbial groups themselves were closely and positively interlinked: bacterial and fungal target gene copies were strongly correlated (r = 0.97, *p* < 0.05), and actinomycete target gene copies were significantly (*p* < 0.05) positively correlated with both bacterial and fungal target gene copies (r = 0.8 for both). Among the physicochemical factors, NH_4_^+^ was significantly (*p* < 0.05) positively correlated with NO_3_^−^ (r = 0.95) and negatively correlated with pH (r = −0.85). As shown in Figure 4b, chitinase (CHI) activity was significantly (*p* < 0.05) positively correlated with the target gene copies of fungi (F) and bacteria (B) (r = 0.8 for both) but not with those of actinomycetes (A). Urease (URE) activity showed no significant correlation with any of the three microbial groups, whereas it was significantly (*p* < 0.05) positively correlated with CHI activity (r = 0.8). Catalase (CAT) activity was significantly (*p* < 0.05) positively correlated with actinomycete target gene copies (r = 0.7) but not with bacterial or fungal copies; it was also significantly (*p* < 0.05) positively correlated with both CHI and URE activities (r = 0.8 for both). Collectively, these results indicate that soil available phosphorus and enzyme activities, particularly chitinase and catalase, are significantly correlated with soil microbial target gene copies.

### 3.5. Multiple Linear Regression Analysis of the Associations Between Soil Pathogen Target Gene Copies and Soil Physicochemical Properties, Enzyme Activities, and Microbial Abundances

As shown in Table 1, the multiple linear regression of *FOC* target gene copies on the seven soil physicochemical factors was highly significant (R^2^ = 1.0000, F = 11,395.49, *p* = 0.0072). Five of the seven factors showed significant (*p* < 0.05) partial effects: SOM (F = 797.97, *p* = 0.023), NH_4_^+^-N (F = 598.18, *p* = 0.026), EC (F = 552.58, *p* = 0.027), AP (F = 221.74, *p* = 0.043), and pH (F = 197.91, *p* = 0.045), whereas NO_3_^−^-N (*p* = 0.143) and AK (*p* = 0.259) had no significant effects. Based on the LMG relative importance metric, SOM explained the largest share of variance (27.24%), which was followed by AK (16.25%), AP (16.13%), and NH_4_^+^-N (13.15%). As shown in Table 2, the regression of FOC target gene copies on the six microbial and enzyme factors was also highly significant (R^2^ = 0.9991, F = 391.74, *p* = 0.0025). Fungal and actinomycete target gene copies both exerted significant (*p* < 0.05) negative partial effects on FOC target gene copies (F = 220.26, *p* = 0.004 and F = 143.26, *p* = 0.007, respectively), and they ranked as the two most important predictors with LMG values of 32.69% and 21.46%. Among the three enzymes, catalase (F = 77.49, *p* = 0.013), urease (F = 31.88, *p* = 0.030), and chitinase (F = 21.66, *p* = 0.043) were all significantly (*p* < 0.05) associated with FOC target gene copies. In contrast, bacterial target gene copies showed no significant effect (*p* = 0.102), despite a relatively high LMG share (22.04%), which suggests that their predictive contribution largely overlaps with that of fungi and actinomycetes. For both models, the Shapiro–Wilk test confirmed that the residuals did not deviate from normality (*p* = 0.543 and *p* = 0.770, respectively). Collectively, these results indicate that fungal and actinomycete target gene copies, together with the three soil enzyme activities, dominate the multivariate explanation of FOC gene copy variation, whereas bacterial target gene copies provide little independent information.

### 3.6. Effects of Different Rotation Regimes on the Abundance of Soil Antagonistic Microorganisms

As shown in Table 3, the proportion of antagonistic fungi was higher in both rotation-crop planting treatments compared with the BB control. The proportion of antagonistic fungi reached 13.3% in the PP treatment and 23.6% in the BP treatment. For antagonistic actinomycetes, the proportion in the BP treatment was 10.0%, which was higher than that in the BB treatment.

### 3.7. Effects of Different Pineapple Cropping Regimes on Banana Plant Height, Stem Diameter, and Leaf Area

**Figure 5:**
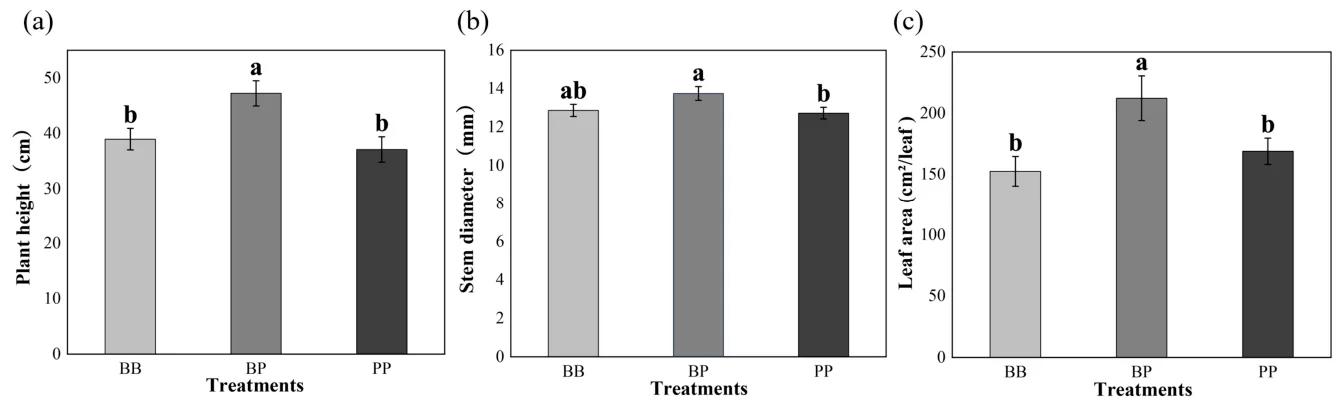
Effects of different rotation patterns on banana seedling growth indicators ((**a**) plant height, (**b**) stem diameter, (**c**) leaf area). Notes: The lowercase letters marked in the figure indicate significant differences among different treatments at the *p* < 0.05 level based on Tukey’s HSD test; the same applies below. BB: Continuous banana monocropping; BP: Single-season pineapple rotation; PP: Long-term pineapple monocropping after continuous banana monocropping.

As illustrated in Figure 5a, compared with the BB treatment, plant height was significantly (*p* < 0.05) increased in the BP treatment, while no significant difference was observed between PP and BB. Additionally, plant height in BP was significantly (*p* < 0.05) higher than that in PP. As shown in Figure 5b, no significant difference in stem diameter was detected between either rotation treatment (BP or PP) and the BB control. Stem diameter in BP was significantly (*p* < 0.05) higher than that in PP. As presented in Figure 5c, leaf area was significantly (*p* < 0.05) higher in the BP treatment than in BB, while no significant difference was found between PP and BB. Leaf area in BP was also significantly (*p* < 0.05) higher than that in PP.

## 4. Discussion

### 4.1. Shifts in Soil Biological, Physicochemical, and Enzymatic Properties Under Rotation Regimes

Crop rotation reshaped the soil’s biological, physicochemical, and enzymatic properties in a coordinated manner. As a key soil-borne pathogen, *F. oxysporum* disrupts the rhizosphere balance and reduces microbial diversity, which ultimately threatens soil health and crop growth [25]. FOC gene copies declined under both rotation treatments, and the decline was greatest under long-term pineapple monocropping; Hong et al. [26] reported a similar pattern. After the crop is switched, host-specific root exudates are gradually depleted, so the pathogen loses its nutritional basis and persists in soil with increasing difficulty [26]. Rotation also raised the target gene copies of total bacteria, fungi, and actinomycetes, as reported by Yuan et al. [18]. Relative to monocropping, rotation changes the root exudate profile, which improves soil properties and strengthens the soil’s natural capacity for disease suppression [27,28]. We also noticed that fungal and bacterial abundances remained higher under single-season rotation than under long-term pineapple monocropping. Two reasons may explain this difference. Under prolonged pineapple monocropping, particular microbial groups may accumulate chemotactically and the community becomes simplified; at the same time, the repeated planting of one crop exhausts specific nutrients, which constrains the microorganisms depending on them. When a new crop is grown for only one season in previously monocropped soil, soil properties and nutrient composition shift rapidly, the established microbial community is broken up, and more diverse niches and nutrient sources become available, all of which favor soil microbial growth and reproduction [29]. Actinomycetes did not follow this trend, because their abundance has been reported to decline once rotation lasts beyond a certain number of years. As pineapple monocropping continues, the soil environment gradually becomes similar to that of continuous pineapple cropping. This convergence weakens the beneficial effect on actinomycetes, so long-term pineapple monocropping and continuous cropping finally reach similar actinomycete levels [30].

Soil physicochemical properties also shifted with the treatments. These properties are key indicators of soil fertility and health, and they directly affect crop root growth [31]. Compared with continuous banana monocropping, long-term rotation-crop monocropping significantly increased soil EC, while single-season rotation had no obvious effect; a similar result was reported by Zhu et al. [32]. One possible explanation is that soluble salts accumulate gradually as pineapple monocropping on former banana soil continues, which disturbs the balance between salt input and output. Soil NH_4_^+^ and NO_3_^−^ are the two nitrogen forms that plants can absorb directly, and they reflect the soil’s immediate nitrogen supply capacity [33]. In this paper, single-season rotation increased NH_4_^+^ only and had no clear effect on NO_3_^−^, whereas long-term pineapple monocropping substantially raised both forms, so the total available nitrogen was higher under multi-season rotation than under single-season rotation. One possible reason is that single-season rotation alleviates the problems caused by monocropping and stimulates microbial activity, which helps banana roots take up more NO_3_^−^, whereas long-term rotation-crop monocropping deteriorates soil properties and microbial activity and thus reduces the crop uptake of available nutrients such as nitrogen [34]. Soil organic matter was likewise higher under single-season rotation than under rotation-crop monocropping. Gou et al. [34] also reported that extended rice-garlic rotation lowers soil organic carbon, which is probably because long-term rotation cropping disrupts soil macroaggregates and accelerates the mineralization and decomposition of native organic carbon so that decomposition eventually outpaces new organic carbon input [35,36].

Additionally, soil enzyme activities are essential for soil nutrient cycling and microbial performance [37]. In this paper, single-season rotation increased catalase and urease activities relative to continuous and long-term rotation-crop monocropping, and short-term rotation has indeed been reported to benefit soil catalase and urease activities [38]. Unlike the previous single crop, short-term rotation introduces a new crop type, which supplies a different nutrient composition and facilitates the proliferation of beneficial soil microorganisms, thereby further enhancing various soil enzyme activities [39]. Soil enzyme activity patterns matched the trends of microbial abundance, which is probably because single-season rotation ameliorates the adverse soil properties left by long-term continuous monocropping and, in doing so, increases enzyme-secreting beneficial microbes. As planting years increase, the microbial community under long-term pineapple monocropping gradually shifts toward a continuous-pineapple structure, and the soil environment deteriorates accordingly, limiting further increases in key enzyme activities [40]. Chitinase is mainly derived from soil actinomycetes, bacteria, and fungi, and it can degrade fungal cell walls to some degree [41]. Here, chitinase activity increased significantly under single-season rotation, which may be explained by the rapid improvement of microbial community structure in continuously cropped soil, where chitinase-secreting microorganisms become more abundant and chitinase activity rises accordingly [37]. No significant chitinase difference was found between long-term rotation-crop and continuous monocropping. A likely reason is that long-term rotation-crop monocropping induces gradual microbial community shifts that eventually resemble continuous rotation-crop cultivation; the soil environment then deteriorates as it does under continuous monocropping, pathogen-inhibiting microorganisms decrease markedly, and chitinase production declines as a result [42].

### 4.2. Relationships Between Soil Pathogen Abundance and Soil Physicochemical Properties, Enzyme Activities, and Microbial Abundance

Soil physicochemical and biological properties together shape soil-borne disease progression, and microbial populations indirectly reflect the effects of soil properties and enzyme activities on pathogen suppression [27]. Correlation analysis showed that FOC gene copies positively correlated with the SOC, available potassium, and available phosphorus. Under long-term continuous monocropping, single-crop root exudates impair microbial community function. They slow organic matter decomposition and reduce nutrient use efficiency. These substances thus accumulate in soil, providing resources for pathogen growth and reproduction [43,44,45]. FOC gene copies also negatively correlated with nitrate nitrogen. Nitrate nitrogen controls soil-borne diseases through multiple mechanisms, including lowering soil pH, inducing host defense responses, and inhibiting FOC spore germination and mycelial spread [46,47,48]. In the multiple linear regression, however, nitrate nitrogen showed no significant partial effect (*p* = 0.144) once the other physicochemical factors were taken into account, and the significant physicochemical predictors of FOC variation were instead SOC, NH_4_^+^-N, EC, AP, and pH. It is worth mentioning that long-term pineapple monocropping (PP) had the highest nitrate nitrogen yet the lowest FOC copies. A likely explanation is that shifting from banana to pineapple modulates soil nitrate through altered root exudation and nitrification, which contributes to pathogen suppression. This inference still requires further validation, since soil nitrogen dynamics are also shaped by fertilizer management, redox conditions, and crop-specific nitrogen preferences. Available phosphorus was negatively correlated with urease activity, and nitrate nitrogen was negatively correlated with chitinase activity. When microbial abundance and enzyme activity are high, crops utilize nutrients more efficiently, so less available phosphorus and nitrate nitrogen accumulate in soil [49,50,51]. Urease activity was also positively correlated with fungal, bacterial, and actinomycetal gene copies (r = 0.57–0.62), although these associations did not remain significant after FDR correction; this trend is still in line with Guo et al. [52]. Most soil urease derives from microorganisms during reproduction and metabolism. Higher total microbial biomass thus directly promotes urease production and accumulation [53,54,55]. Rotation increased both microbial abundance and urease activity. Rotation systems can therefore alleviate damage to soil physicochemical and biological properties from continuous monocropping. They also promote beneficial microbial groups that produce urease. The multiple linear regression model further showed that soil organic matter, available potassium, available phosphorus, and ammonium nitrogen each accounted for >13% of the explained FOC variation. This supports that physicochemical factors indirectly determine pathogen population thresholds by shaping microbial habitats [12,25]. These factors are likely the most important physicochemical drivers of pathogen temporal shifts. Fungal gene copies accounted for >30% of the explained variation, bacterial and actinomycete copies for about 22% and 21%, and urease activity for around 15%. In this model, fungal and actinomycete gene copies together with the three enzyme activities all showed significant (*p* < 0.05) partial effects on FOC gene copies, whereas bacterial gene copies did not (*p* = 0.102). These results support Yuan et al. [18] and Wang et al. [56]. Fungal community shifts serve as the chief biological mediator of pineapple-rotation disease suppression. Das et al. [57] likewise noted that chitinase activity reliably indexes rhizosphere fungal antagonism. BP generally showed more favorable soil biological properties than PP, yet PP maintained lower FOC copies. Host-specific resource depletion may therefore suppress pathogens through a pathway distinct from and complementary to microbiome-mediated suppression.

### 4.3. Factors Influencing Antagonistic Microorganisms Under Different Banana–Pineapple Rotation Regimes

Beneficial microorganisms with pathogen-inhibiting activity are key functional groups driving the disease-suppressive function of soil, and their abundance directly determines the pathogen suppression capacity of soil [58]. In this paper, both BP and PP significantly increased microbial target gene copies in continuously cropped soil. Pathogen antagonism assays showed that the proportions of both antagonistic fungi and antagonistic actinomycetes were significantly increased in the single-season rotation treatment, and the proportion of antagonistic fungi in single-season rotation was higher than that in long-term continuous cultivation of the rotation crop and long-term continuous monocropping. This result is similar to the findings of Zhang et al. [59], who reported that single-season rotation improved the microbial community structure in continuously cropped soil with an increased proportion of fungi antagonistic to pathogens. It can be inferred that the results of this paper may be attributed to the fact that single-season rotation promotes the abundance of chitinase-secreting microorganisms, which degrade the cell walls of fungi including pathogens, thereby reducing pathogen abundance [57]. In contrast, the proportion of antagonistic microorganisms in long-term continuous cultivation of the rotation crop was significantly lower than that in single-season rotation, which may be due to the deterioration of soil properties under long-term continuous cultivation of the rotation crop. Meanwhile, no significant difference in the proportion of antagonistic actinomycetes was observed between long-term continuous cultivation of the rotation crop and long-term continuous monocropping in this paper. This result is consistent with the findings of Liu et al. [60], who reported that long-term continuous soybean monocropping (13 years) had similar effects on regulating microbial abundance, diversity, and community composition to those of short-term maize–soybean rotation (5 years). This may be because with the extension of planting duration, soil physicochemical and microbial properties gradually deteriorate, and persistent environmental filtering leads to a directional convergence of microbial communities, resulting in no significant difference in the proportion of antagonistic actinomycetes between long-term continuous cropping and continuous cultivation of the rotation crop. Therefore, single-season rotation can increase the proportion of antagonistic microorganisms in rotated soil, which significantly enhances the disease-suppressive capacity of soil, while the promoting effect of continuous cultivation of the rotation crop in this regard is diminished.

### 4.4. Factors Influencing Banana Seedling Plant Height, Stem Diameter, and Leaf Area Under Different Rotation Regimes

Plant height, stem diameter, and leaf area are all reliable indicators reflecting plant growth status: plant height reflects a plant’s vertical growth potential and developmental stage, stem diameter indicates the strength of a plant’s mechanical support and the transport efficiency of its vascular tissues, and leaf area represents the plant’s light interception capacity and potential photosynthetic productivity [61]. In this paper, single-season rotation significantly increased banana plant height, stem diameter, and leaf area, whereas no significant differences in these growth indicators were observed between long-term continuous monocropping and continuous cultivation of the rotation crop. These results differ from the earlier findings of Zeng et al. [62], who reported that growth indicators such as plant height and stem diameter continued to increase as the planting duration of rotation-suitable crops extended from 1 year to 3 years. The discrepancy between the two studies likely lies in the planting duration: within short periods of 1 to 3 years, prolonging the cultivation of rotation-suitable crops helps gradually alleviate continuous cropping obstacles, whereas in this paper, the rotation-suitable crop had been planted for more than five years. When a single rotation-suitable crop is grown repeatedly over such a long period, the soil properties gradually become similar to those of fields under continuous monocropping of that same crop, and crop growth is finally inhibited in much the same way as in continuous monocropping soil. Single-season rotation works differently. It can rapidly mitigate the problems caused by continuous cropping, because it improves the microbial community and the physical and chemical properties of continuously cropped soil, which together promote better crop growth [63]. Overall, our findings indicate that soil under single-season rotation is more effective at supporting the healthy growth of subsequent continuous crops. Long-term cultivation of the rotation-suitable crop, however, can also deteriorate soil properties and is thus unfavorable for the growth of continuous crops.

## 5. Conclusions

Field trials conducted over the long term were paired with pot validation assays to contrast continuous banana monocropping against rotation-suitable crops grown for differing durations. The cultivation regimes of rotation-suitable crops significantly reduced soil FOC target gene copies, with long-term cultivation showing greater effects of pathogen suppression in the absence of banana hosts, but it aggravated soil acidification and salt accumulation without improving microbial abundance or enzyme activities. And single-season rotation, in contrast, increased the target gene copies of total bacteria, fungi, and actinomycete, enhanced catalase, urease, and chitinase activities, elevated the proportions of antagonistic microbes, and promoted the growth of banana seedlings. Model analysis identified soil organic matter, available potassium, available phosphorus, and ammonium nitrogen among the physicochemical factors, together with fungal and actinomycete target gene copies and the activities of catalase, urease, and chitinase, as key FOC regulators. Taken together, these findings may help elucidate how pineapple cultivation in banana-monocropped soil suppresses the pathogen number by reshaping the soil physicochemical and biological properties, and they suggest single-season rotation as a more practical choice than prolonged pineapple cropping for mitigating *Fusarium* wilt risk and continuous cropping obstacles. Notably, as both banana and pineapple are perennial crops, the generalizability of these findings to annual or seasonal cropping systems requires further evaluation.

## 6. Limitations

Several limitations of this paper should be acknowledged. To begin with, microbial abundance was estimated from qPCR-based 16S and ITS rRNA gene copy numbers. Since the rRNA gene copy number varies across taxa, the measured copy numbers may not strictly reflect absolute cell numbers, and shifts in community composition could contribute to apparent changes in gene copy abundance independent of actual changes in cell density. The qPCR results herein should thus be interpreted as relative estimates of microbial gene abundance for comparing treatment effects rather than as any absolute quantification of microbial biomass or cell numbers. A second limitation concerns disease assessment: root staining and the direct evaluation of banana Fusarium wilt incidence and severity were not performed, so the observed reduction in soil FOC abundance cannot be directly equated with disease suppression, and the link between pathogen inhibition and actual disease control requires further field validation. Finally, with only three biological replicates per treatment, the statistical power and robustness of the multiple linear regression and correlation analyses may be limited; these results should therefore be regarded as exploratory rather than confirmatory, and the identified key drivers await validation with larger sample sizes.

## Figures and Tables

**Table 1:** Multiple linear regression of *FOC* gene copies on soil physicochemical factors (n = 9).

Factors	Df	F	*p*	r	Relative Importance (%)
EC	1	552.58	0.027 *	−0.712	10.92
pH	1	197.91	0.045 *	0.279	4.88
NH_4_^+^-N	1	598.18	0.026 *	−0.720	13.15
Available P (AP)	1	221.74	0.043 *	0.657	16.13
NO_3_^−^-N	1	19.01	0.144	−0.686	11.43
SOM	1	797.97	0.023 *	0.974	27.24
Available K (AK)	1	5.38	0.259	0.792	16.25
Model summary	R^2^ = 1.0000; Adjusted R^2^ = 0.9999; F = 11,395.49; *p* = 0.0072				
Model diagnostics	AIC = −105.16; BIC = −103.58; Shapiro–Wilk W = 0.936 (*p* = 0.543)				

Note: All seven physicochemical factors were entered simultaneously. * *p* < 0.05. r = simple Pearson correlation coefficient with FOC gene copies. Relative importance was quantified by the LMG metric (averaged over all predictor orderings).

**Table 2:** Multiple linear regression of FOC gene abundance on soil microbial and enzyme factors (n = 9).

Factors	Df	F	*p*	r	Relative Importance (%)
Fungi (F)	1	220.26	0.005 *	−0.316	32.69
Actinomycetes (A)	1	143.26	0.007 *	−0.101	21.46
Bacteria (B)	1	8.31	0.102	−0.220	22.04
Urease (URE)	1	31.88	0.030 *	0.188	14.94
Chitinase (CHI)	1	21.66	0.043 *	−0.114	5.81
Catalase (CAT)	1	77.49	0.013 *	0.040	3.06
Model summary	R^2^ = 0.9991; Adjusted R^2^ = 0.9966; F= 391.74; *p* = 0.0025				
Model diagnostics	AIC = −69.21; BIC = −67.83; Shapiro–Wilk W = 0.957 (*p* = 0.770)				

Note: All six microbial/enzyme factors were entered simultaneously. * *p* < 0.05. r = simple Pearson correlation coefficient with FOC gene copies. Relative importance was quantified by the LMG metric (averaged over all predictor orderings).

**Table 3:** Antagonistic microorganisms ratio.

Treatments	Antagonistic Fungi	Antagonistic Actinomycetes
BB	11.8%	2.0%
PP	13.3%	2.0%
BP	23.6%	10.0%

Notes: BB: Continuous banana monocropping; BP: Single-season pineapple rotation; PP: Long-term pineapple monocropping after continuous banana monocropping.

## Data Availability

The data presented in this paper are available on request from the corresponding author. The data are not publicly available due to privacy.
